# Determinant Factors of Public Acceptance of Stress Management Apps: Survey Study

**DOI:** 10.2196/15373

**Published:** 2019-11-07

**Authors:** Jennifer Apolinário-Hagen, Severin Hennemann, Lara Fritsche, Marie Drüge, Bernhard Breil

**Affiliations:** 1 Institute of Occupational, Social and Environmental Medicine Faculty of Medicine, Centre for Health and Society Heinrich Heine University Düsseldorf Düsseldorf Germany; 2 Department of Clinical Psychology, Psychotherapy and Experimental Psychopathology Institute of Psychology University of Mainz Mainz Germany; 3 Department of Health Psychology Faculty of Psychology University of Hagen Hagen Germany; 4 Psychotherapy Research, Department of Clinical Psychology Institute of Psychology University of Zurich Zurich Switzerland; 5 Faculty of Health Care Niederrhein University of Applied Sciences Krefeld Germany

**Keywords:** mental health, eHealth, mHealth, attitude to computers, acceptability of health care, stress, psychological, mobile apps

## Abstract

**Background:**

Chronic stress is a major public health concern. Mobile health (mHealth) apps can help promote coping skills in daily life and prevent stress-related issues. However, little is known about the determinant factors of public acceptance of stress management in relation to preferences for psychological services.

**Objective:**

The aim of this survey study was to (1) assess determinant factors of public acceptance (behavioral use intention) of stress management apps based on an adapted and extended version of the Unified Theory of Acceptance and Use of Technology (UTAUT) model and (2) explore preferences for mHealth apps compared with other mental health services.

**Methods:**

Using convenience sampling, participants completed a multiscale 54-item Web-based survey. Based on significant correlations with acceptance, hierarchical stepwise regression analysis was performed within three blocks: (1) background and stress-related control variables, (2) beliefs and attitudes toward using mHealth, and (3) the core UTAUT determinants. The preference for mHealth apps in comparison with nine other mental health services (operationalized as readiness to use) was analyzed using paired *t* tests.

**Results:**

Of 141 participants, nearly half (69/141, 48.9%) indicated prior mHealth use. Acceptance of stress coping apps was moderate (mean 3.10, SD 1.03, range 1-5). Hierarchical stepwise regression including four of 11 variables (R^2^=.62; *P*=.01, f^2^=1.63) identified positive attitudes toward using mHealth for stress coping (beta=0.69, *P*<.001, 46% R^2^ increase above block 1, f^2^=0.85), skepticism/perceived risks (beta=−0.14, *P*=.01, f^2^=0.16), and stress symptoms (beta=0.12, *P*=.03, f^2^=0.14) as significant predictors of acceptance. UTAUT determinants added no predictive contribution beyond attitudes (all *P*>.05, R^2^ increase of 1%), whereas post hoc analysis showed significant R^2^ increases of attitudes and skepticism/perceived risks beyond UTAUT determinants (all *P*<.001, R^2^ increase of 13%). The readiness to use apps was equivalent to or significantly higher than most service types, but lower than information websites.

**Conclusions:**

Attitudes may be at least as predictive for the acceptance of stress management apps as for more elaborated outcome beliefs. Efforts aimed at improving the public adoption of mHealth could put more emphasis on the pleasant aspects of app use, address misconceptions, offer stress screening tools on health websites, and increase options to try high-quality apps.

## Introduction

### Background

Chronic stress represents a tremendous health risk [1-3] and is a key contributor to the global burden of mental illness, which results in high economic costs on a societal level [4]. Therefore, from a public health perspective, it is vital to invest in the prevention of stress-related health problems. In this paper, stress is to be understood according to the Transactional Stress Model by Lazarus and Folkman [5], according to which subjective stress and coping appraisals caused by an event can result in further problem-focused or emotion-focused coping strategies.

These strategies are the centerpiece of efficacious cognitive behavioral and multimodal stress management interventions, which are commonly provided in group settings [6]. Beyond group interventions, e-mental health services that can be delivered via mHealth apps may increase public access to interventions for the prevention of mental health problems [7]. Utilization rates in target groups in the field of workplace health promotion may also increase [8-10] by providing effective occupational e-mental health interventions for employees [11-13].

Meta-analyses have demonstrated that high-quality mental health apps are efficacious in reducing the symptoms of anxiety [14], depression [15], and stress [16]. There are evidence-based digital stress management programs for nonclinical target groups, such as employees (eg, GET.ON [17-20]) and university students (eg, StudiCare [21-23]).

Common content or behavior change techniques of available stress management apps involve problem-focused strategies, such as time management, goal setting, and planning social support. Emotion-focused strategies often include relaxation techniques, such as breathing exercises, mindfulness, or meditation and autogenic training [24].

Although more than 10,000 mental health apps are publicly available, very few have been evaluated scientifically [25]. Health policy is necessary to ensure the structural requirements for the dissemination of high-quality, safe, and effective apps. To date, only a few stand-alone mHealth apps have been evaluated in randomized controlled trials (RCTs) and meet the criteria for becoming prescribable in medical contexts [26].

In 2018, the German National Association of Statutory Health Insurance Funds made it possible to cover the costs of certified digital self-help programs for insured persons [27]. Furthermore, with the recently passed draft of the Digital Healthcare Act (Digitale-Versorgung-Gesetz [28]), the German Federal Ministry of Health set the course for the prescription of quality-approved mHealth apps.

Despite increasing efforts to promote the diffusion of e-mental health worldwide, there is a remarkable discrepancy between the interest in and real-world uptake of mental health apps [29,30]. A comprehensive understanding of user characteristics, as described in the behavior change model for internet interventions [31] (eg, demographic and health-related variables as well as attitudes and beliefs), represents an essential first step to create persuasive digital interventions [31-33].

#### Assessment of the Acceptance of Stress Management Apps

Hennemann et al [34] acknowledged the confounding with intervention satisfaction as a major methodological weakness of the commonly practiced retrospective assessment of acceptance of e-mental health services, which does not allow for exploring genuine attitudes or reasons for use or nonuse.

Predictive models of acceptance of information technology, such as the Unified Theory of Acceptance and Use of Technology (UTAUT) [35], operationalize acceptance as the strength of one’s behavioral intention to use a novel technology [36-38].

Given that the assessment of technology acceptance is in many ways context-sensitive [34], the operationalization of UTAUT predictors has to be adapted to the respective type or purpose of the intervention [39], health outcome, or target population [40]. A growing body of research has used the UTAUT framework to investigate eHealth acceptance in various contexts, such as disease management apps for chronic illness [41,42] and Web-based interventions for depression [43,44], chronic pain [45], and occupational stress [34,46]. A low-to-moderate acceptance was indicated across all studies.

In view of our scope on mHealth for health promotion and stress reduction, we expected a moderate or slightly higher acceptance of mHealth for stress coping in a sample of internet users compared with surveys of patients in health care settings.

### Determinants of the Acceptance of Stress Management Apps

According to the generic UTAUT model, performance expectancy (eg, perceived usefulness), effort expectancy (eg, ease of use), and social influence (eg, subjective norm) are predictors of the intention to use an innovative technology, whereas facilitating conditions (eg, perceived behavioral control) and behavioral intention are hypothesized as direct determinants of actual use [35].

Generally, most research on the UTAUT model point to performance expectancy as the strongest driver of technology acceptance across different contexts and innovations [47-49], including eHealth services [34,46,50-52].

Beyond core UTAUT determinants, several additional predictors of technology acceptance have been suggested, particularly attitude [48], which was excluded as a key determinant from the UTAUT model [35].

Attitudes can be defined as cognitive or affective evaluative judgments of psychological objects, for instance, in terms of one’s positive or negative feelings toward performing a behavior [36,53-55]. These attitudes are often associated with outcomes of health interventions [56,57]. As positive perception of and satisfaction with using a health technology [58], attitudes have been proposed as an essential precondition for the adoption of e-mental health services [59-62]. Recent meta-analyses support the integration of attitudes and UTAUT beliefs in technology acceptance models [48] and the way that beliefs about the usefulness and ease of use strongly influence attitudes, which positively affect behavioral intentions to use mHealth apps [63].

In turn, negative attitudes could play a more relevant role for the poor uptake of e-mental health interventions than structural barriers [34]. Negative attitudes can involve skepticism and perceptions of risks of e-mental health interventions [61]. For example, data security or privacy concerns represent common reasons for not using the internet or mobile phones for mental health purposes [30,34,64-66], whereas anxiety toward using technology can negatively affect behavioral intention to use mHealth [50].

Hence, we assumed a positive influence of attitudes (as a driver) and negative influences of skepticism and related negative beliefs (as barriers) on the acceptance of using mHealth for stress coping.

Also, low awareness of mHealth apps and deficient mHealth literacy represent barriers to adoption [67,68]. Studies indicate a positive influence of experience with health-related internet or mobile phone use [32,34,46] on the acceptance of e-mental health services [32] and the real-world adoption of mHealth apps [69]. Although mHealth app users were found to be younger [70,71], more highly educated, and healthier than nonusers (eg, [71]), findings regarding demographic variables on the acceptance of e-mental health services are less consistent. More favorable views on e-mental health services were found among young adults [34,62,72], women [62,73], and adults with higher education [34,62,72,73], whereas other studies found no gender difference [34,64].

Remarkably, the motivating influence of current needs (eg, for support in stressful situations) on intentions to use e-mental health services has not been consistently clarified. On the one hand, there is evidence for an association between stress perceptions and attitudes toward using e-mental health treatments [33,74] and a higher interest in using stress management apps [75]. On the other hand, there is evidence for “digital stress” caused by online multitasking and overload (eg, [76]). A recent study showed an association between intense media use for social networking and relaxation/entertainment and emotional stress [77]. Also, stress due to permanent online availability has been demonstrated as a barrier for inpatients’ acceptance of Web-based aftercare [34].

In view of the inconsistent or limited findings on the role of background variables, as well as stress and coping appraisals on the acceptance of mHealth, we proposed influences of these constructs on acceptance in terms of control variables.

Another influencing factor for the adoption of mHealth apps could be the way they are described to consumers in app stores. Huang and Bashir [78] found positive associations of information cues (reviews, ratings in app stores) with the number of downloads of mental health apps for anxiety. In contrast, Healey et al [79] identified no impact of expert and user testimonials on registrations for an unguided, Web-based depression intervention (MoodGym). Another RCT investigating public attitudes toward e-mental health treatments [59] observed a positive influence of information supplemented with scientific claims on an exemplary e-mental health service on attitudes, but not on intentions of use. In clinical contexts, there is also evidence of a positive impact of psychoeducational information on patients’ acceptance of e-mental health treatments [43,44]. However, the heterogeneous evidence base demonstrates the need for further research on the relevance of information cues in app descriptions for the uptake in the relevant target.

Based on what is already known from other contexts, we expected a positive influence of scientific claims on stress coping apps on general acceptance.

#### Preference for and Readiness to Use Mobile Health for Stress Coping

The outcomes of psychological services are associated with individual preferences [80]. Concerning mHealth, a German panel survey showed that 53.29% of participants were not considering using apps for consultation or treatment [81]. Moreover, research points to a clear public preference for face-to-face treatment over e-mental health treatment services [32,34,62,65,82,83]. A study on the public acceptability of e-mental health treatments found the lowest likelihood for using mHealth apps, whereas the readiness to use Web-based interventions and self-help books were equivalent [83]. Another study found differences in the likelihood of using traditional services (eg, psychologist) and digital services (eg, information website) between people who either preferred or did not prefer e-mental health, but not for self-help books or medical treatment (general practitioner, prescribed medication) [82]. In addition, studies conducted in Germany showed a high interest in using health information websites and a low-to-moderate acceptance of mHealth apps and Web-based programs for dealing with stress [34,84].

In contrast to surveys, real-world self-help activities can hardly be condensed into a forced-choice format because services are often used simultaneously (eg, app and website search). Hence, it would be interesting to learn more about patterns of preferences for apps versus other available or prototypical mental health services. This would help to integrate findings on mHealth acceptance in a greater practice-oriented context and enable practitioners to tailor their recommendations of mental health services to clients’ needs and preferences.

Based on these considerations in the context of health promotion, we assumed a preference for using digital self-help services (apps, websites) and psychological support over medical help for dealing with everyday stress.

### Goals of This Study

The primary aim of this survey study was to assess the determinants of public acceptance of mHealth stress coping apps in an online sample of adults. We expected a positive influence of mHealth-related attitudes and beliefs and a negative influence of skepticism or perceived risks on the acceptance of stress management apps. Furthermore, in direct relation to the primary outcome, we were interested in the potential differences in acceptance and its determinants based on information cues in the description of a sample stress coping app (either with or without scientific claims).

Another purpose was to assess preferences for mHealth apps compared with other psychological services for dealing with stress to set the main findings in a greater context of the general readiness to use stress prevention services.

## Methods

### Study Design and Data Collection

Data for this cross-sectional 54-item survey applying a descriptive predictive research design were collected anonymously at the University of Hagen in Germany between May 25, 2017, and June 16, 2017, using Unipark (Enterprise Feedback Suite survey, version summer 2017, Questback, Germany). All items were only available in the German language. The average completion time was 10 to 15 minutes.

Participants were informed about the study’s objective and procedure beforehand (eg, health psychological research project in terms of a survey the general acceptance of and preferences for digital solutions for stress reduction) and were required to give an informed consent online (click-to-agree) following the recommendations of the German Psychological Association [85].

As part of a research agenda with different subprojects, this survey was the pilot study for a follow-up project with an equivalent objective and methodology (public acceptance of certified stress management programs), which has received ethical approval by the recently founded institutional review board/local EC of the new Faculty of Psychology at the University of Hagen, Germany (reference: EA_85_2019).

To establish a consistent understanding of the type of mHealth under study (stress management app), participants were presented a brief description of a sample or hypothetical app (similar to plain lay product information for consumers on websites or in app stores) before answering acceptance-related questions. The hypothetical app in our study was described as a digital solution that helps consumers cope with stress in everyday life or at work.

The text for the description of the sample app was adapted and modified from the German website of the digital program StudiCare Stress/Fernstudierende [23] that provided information relevant for study participation in an evidence-based digital stress coping program for distance-learning students in 2017. The idea behind describing a hypothetical app was to avoid advertising a specific app and adding a potentially confounding influence of experience with the use of real apps. The information for both groups was provided in relation to this hypothetical stress coping app (using two vignettes, as shown in Textbox 1). Therefore, participants were aware of being asked to imagine which expectations they would have regarding a fictional app, which was later confirmed by feedback from participants through online contact. The approach of implementing vignettes to describe prototypical or exemplary services in this research field is established and has been applied in several other studies (eg, [43,59,82,86-88]).

To assess whether scientific claims would contribute to greater acceptance compared with basic information, participants were randomly assigned (50:50 allocation) to one of two information groups that contained the description of the hypothetical app either with or without supplemented scientific claims (Textbox 1).

**Randomized subsection of the survey with text from a sample stress coping app with or without supplemented information on scientific claims.**

- Both information groups 1 and 2 received the same following basic information (basic vignette):

*“Stress can be triggered by different situations in daily life. If stress becomes a permanent condition, it can seriously endanger one’s physical and mental health. ‘COPE—Computer-gestützte, Online-basierte personalisierte Entspannung* [*Computer-aided, online-based, personalized relaxation*]*,’ is an app that helps you to better cope with stress, especially in everyday/working life, and to support you flexibly in terms of time.”*

- Scientific claims were only visible for the participants randomized to group 2 (supplemented vignette):

*“Efficacy studies have shown that ‘COPE’ has an excellent effect and reduces stress sensations even after one year of training. There are also reports of fewer depressive symptoms, emotional exhaustion, and anxiety. The app was developed by leading international scientists in the field of stress and e-mental health research.”*

- Finally, both information groups received this instruction:

*“Imagine if you would own this app—what expectations would you have?”*

### Participants and Recruitment

Using convenience sampling, an online sample of German-speaking adults was recruited via social media websites (eg, Facebook) and personal contacts of the study team. Exclusion criteria were age younger than 18 years and a decline or withdrawal of consent. A summary of aggregated findings was offered as compensation for participation. Participants could contact the study team via email in case of having questions or any feedback.

An a priori power analysis using G*Power [89], version 3.1 (linear multiple regression, *F* tests, fixed model, *R*^2^ increase) resulted in a required sample size of at least N=135 to determine a minimum moderate effect size of f^2^=0.15 [90] (alpha=.05, power=.85; noncentrality parameter=20.25, critical *F*_11,123_=1.87). The effect size was justified based on similar research on e-mental health acceptance [34,46].

### Measures

#### Primary Outcome: Determinants of Acceptance of Stress Management Apps

**Figure 1:**
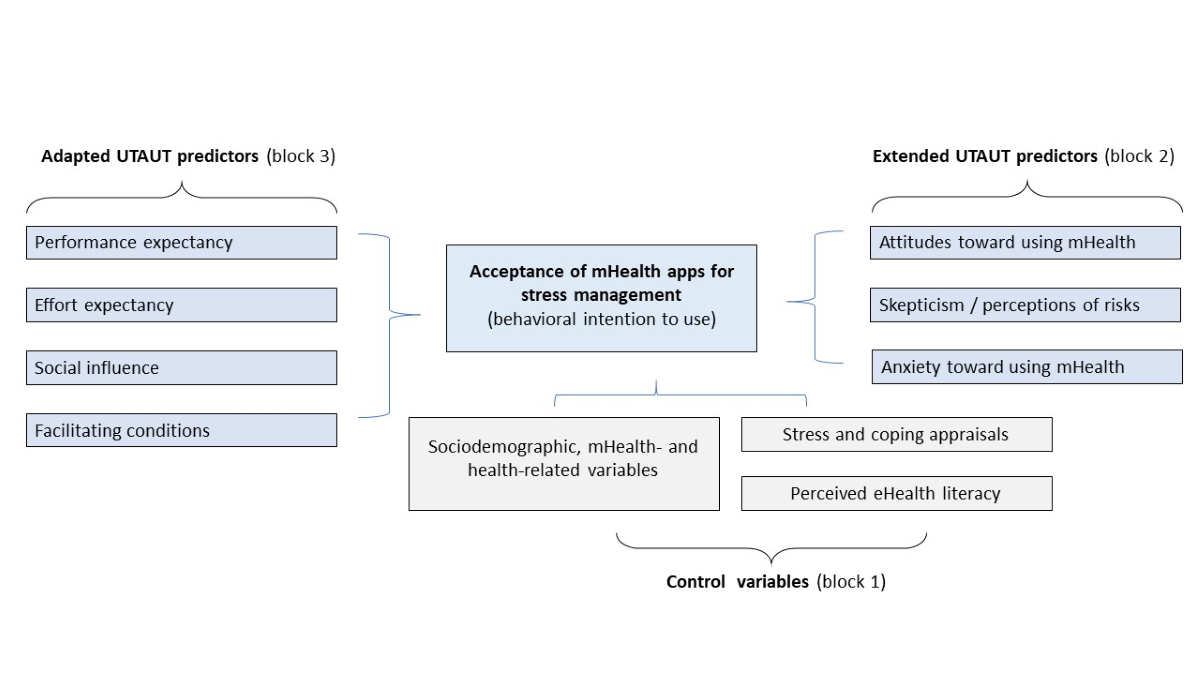
Conceptual study model using an adapted and extended UTAUT model for the assessment of acceptance of mHealth apps for stress coping. mHealth: mobile health; UTAUT: Unified Theory of Acceptance and Use of Technology.

Measures of the adapted and extended predictive mHealth acceptance study model (Figure 1) are presented in Table 1. Multimedia Appendix 1 (Table S1) contains a full overview of the content and reference studies of UTAUT-related items; we slightly adapted to the context of mHealth for stress coping based on face validity.

**Table 1:** Summary of constructs, measures, and scales for the assessment of determinants of acceptance of mobile health for stress coping.

Construct		Measure	Items, n	Cronbach alpha
**Dependent variable**				
	Acceptance of mHealth^a^ for stress management	UTAUT^b^: behavioral use intention^c,d,e^	3	.88
**Core UTAUT determinants**				
	Performance expectancy	UTAUT	4	.91
	Effort expectancy	UTAUT	4	.84
	Social influence	UTAUT	3	.82
	Facilitating conditions	UTAUT	2	.86
**Extended UTAUT determinants**				
	Attitudes toward use of technology (positive affect toward using apps)	UTAUT^f^	4	.90
	Anxiety toward use of mHealth	UTAUT	4	.83
	Skepticism and perceived risks (negative attitudes)	APOI^c,e,g^	3	.67
**Control variables**				
	eHealth literacy	G-eHEALS^e,h^	8	.91
	Permanent smartphone availability	Self-constructed (single item)^e,i^	N/A^j^	N/A
	Stress due to overload (past 3 months)^i,k^	SCI: stress scales^l,m^	7	.76
	Stress symptoms (severity, past 6 months)^i,l^	SCI: stress scales^k^	13	.86
	Positive thinking^k,n^	SCI: coping scales^k,n^	4	.71
	Active coping^k,n^	SCI: coping scales	3	.87
	Social support^k,n^	SCI: coping scales	4	.88
	Cigarettes and alcohol consumption^k,n^	SCI: coping scales	4	.74
	Demographic/descriptive variables	Age (metric), gender, experience with using a smartphone (yes/no; filter question: frequency), educational level, suffering from a chronic illness or enduring/recurrent complaints for more than 3 weeks (yes/no; filter question: category of illness), experience with use of any kind of mHealth app (yes/no; filter questions: frequency and duration of use), awareness of and experience with internet-based psychotherapy (each with 1 item; yes/no)^o^	N/A	N/A

^a^mHealth: mobile health.

^b^UTAUT: Unified Theory of Acceptance and Use of Technology.

^c^Adapted to mHealth for stress management/coping (Multimedia Appendix 1, Table S1).

^d^German Unified Theory of Acceptance and Use of Technology (GUTAUT) measure for Web-based aftercare by Hennemann et al [34], which the test authors developed based on prior work [43-45,91].

^e^Assessed on a 5-point Likert scale ranging from 1 (fully disagree) to 5 (fully agree).

^f^Adapted from the original UTAUT questionnaire by Venkatesh et al [35], dropped scale in the final UTAUT model.

^g^Assessed with three suitable items of the 4-item subscale “skepticism and perception of risks” of the Attitudes toward Psychological Online Interventions questionnaire (APOI) [61].

^h^Measured using the 8-item German eHealth literacy scale (G-eHEALS) [92].

^i^Based on prior research [34], we constructed a single-item scale (“Do you feel stressed when you are always available via your mobile phone or smartphone?”).

^j^N/A: Not Applicable.

^k^We used two scales (20 items) out of five stress scales (originally 34 items) and further 15 items from four out five coping-scales (originally 20 items) of the German 54-item/10-scale Stress and Coping Inventory (SCI) by Satow [93]. The SCI measures everyday stress perceptions in different areas of life and general coping strategies. It is possible to select scales of interest instead of using the full instrument.

^l^The 7-item-scale SCI (Stress and Coping Inventory)-stress subscale [93] “stress due to overload” related to seven events (eg, item 1: debts or financial issues) concerning the past 3 months was assessed on a 7-point Likert scale ranging from 1 (not overloaded) to 7 (very overloaded).

^m^The 13-items SCI-stress subscale [93] “stress symptoms” covered physical and psychological stress sensations (eg, item 1: “I sleep badly”) concerning the past 6 months was assessed on a five-point Likert scale ranging from 1 (fully disagree) to 5 (fully agree).

^n^Of the coping-scale of the SCI [93], we included four of five subscales, which we assessed on a four-point Likert scale ranging from 1 (fully disagree) to 4 (fully agree). “Active coping” was assessed with three items (originally four items). The scale “support in religion” was dismissed due to questionable relevance.

^o^We evaluated the awareness of and experience with internet-based psychotherapy, each with one item (yes/no). These questions were contributed by the first author to the German Socio-Economic Panel Innovation Sample in the fall 2016 wave [94].

#### Secondary Outcome: Preference for and Readiness to Use Mobile Health for Stress Coping

Based on a help-seeking questionnaire [95] and research on “e-preference” [82,87], the readiness or likelihood to use mHealth apps (strength of preferring mHealth apps over other services) was assessed with a self-constructed 10 item-scale on a five-point response scale ranging from 1 (very unlikely) to 5 (very likely). The question was: “If you would feel distressed, how likely would you use the following services?” The service types were as follows: app versus information website, online self-help training, online counseling, self-help literature, psychologist, psychiatrist, general practitioner (GP), prescribed medication, and on-site group training (face-to-face). Cronbach alpha was good (Cronbach alpha=.80).

### Statistical Analysis

Only completed surveys were entered in the data analysis using SPSS version 24 (IBM Analytics). Based on prior research [34], the mean score of acceptance was categorized as low (1-2.34), moderate (2.35-3.67), or high (3.68-5). The Stress and Coping Inventory (SCI) [93] is not designed as a diagnostic instrument; therefore, no cut-off scores or indexes for stress outcomes are provided.

Following significant zero-order correlation testing, predictors of acceptance were selected to enter a hierarchical stepwise regression analysis. Based on theoretical considerations (eg, [35,36,53-55]) and empirical research (eg, [34,46,61]), we chose three blocks for the stepwise order for entering of predictors. Block 1 contained sociodemographic, mHealth-related variables, and stress-related variables (control variables); block 2 contained attitudes and beliefs related to mHealth (UTAUT extension regarding the affective component; *R*^2^ increase beyond control variables); and block 3 contained the core UTAUT determinants (elaborated beliefs of classic UTAUT; *R*^2^ increase after accounting for the influence of attitudes).

Differences in mean scores for acceptance and its determinants between the two information groups (see Textbox 1) were assessed using *t* tests or Welch *F* tests in case of variance inhomogeneity, respectively.

To assess the preference for mHealth apps for stress coping, differences between mean scores of the likelihood of future use of mHealth apps compared with nine other mental health service types were analyzed using paired *t* tests. Effect sizes were classified based on Cohen’s criteria [90,96]. The significance level for the hypotheses was alpha<.05.

## Results

### Sample Characteristics

Descriptive data on the 141 participants are presented in Table 2. Multimedia Appendix 2 (Table S2 ) contains an overview of self-reported chronic complaints, which were most often upper or lower back pain with 14.2% (20/141).

**Table 2:** Sample characteristics (N=141).

Variables			Participants
**Gender, n (%)**			
	Female		86 (61.0)
	Male		55 (39.0)
	Other		0 (0)
**Age (years)**			
	Mean (SD)		34.84 (11.09)
	Median (range)		31.00 (19-76)
**Education** **level, n (%)**			
	No certificate of education (pupil or left school without certificate)		4 (2.8)
	Certificate of secondary education^a^		6 (4.3)
	General certificate of secondary education^b^		21 (14.9)
	Advanced technical college entrance qualification^c^		6 (4.3)
	General qualification for university entrance^d^		17 (12.1)
	University degree (bachelor level)		42 (29.8)
	University degree (master level)		41 (29.1)
	Postdoctoral degree (doctorate or habilitation)		4 (2.8)
**Stress- and technology-related variables**			
	Having chronic complaints, n (%)		41 (29.1)
	Smartphone use (familiarity with use), n (%)		136 (96.5)
	**mHealth^e^ app use experience (filter question), n (%)**		
		No	71 (51.1)
		Yes	69 (48.9)
	**Frequency of mHealth app use, n (%)**		
		Daily	15 (10.6)
		Several times a week	14 (9.9)
		Weekly	4 (2.8)
		Several times a month	11 (7.8)
		Once a month or less	25 (17.7)
	**Duration of mHealth app use, n (%)**		
		More than 2 years	37 (26.2)
		Less than 2 years	32 (17.4)
	**Awareness of Internet therapies (filter question), n (%)**		
		Yes	30 (21.3)
		No	111 (78.7)
	**Prior use of internet therapies, n (%)**		
		Yes	5 (3.5)
		No	25 (17.7)

^a^German “Hauptschulabschluss” as basic school qualification.

^b^German secondary school level I certificate (“Mittlere Reife”).

^c^German “Fachhochschulreife” or “Fachabitur”.

^d^German “Allgemeine Hochschulreife” (“Abitur” or A-Level).

^e^mHealth: mobile health.

### Preliminary Analyses

Acceptance of using mHealth for stress coping was moderate on average (mean 3.10, SD 1.02; range 1-5). Nearly half of participants could be categorized as reporting a moderate (46.8%, 66/141) acceptance; 29.1% (41/141) reported a low acceptance and 24.1% (34/141) reported a high acceptance.

Based on significant zero-order correlations with acceptance, 11 of 25 variables were selected for the hierarchical stepwise regression analysis (Textbox 2). The highest correlations with acceptance were found for attitudes toward using mHealth (*r*=.77) and performance expectancy (*r*=.64), as shown in Multimedia Appendix 2 (Table S3).

**Predictors of mobile health (mHealth) acceptance investigated in the stepwise regression analysis.**

The order for the stepwise entering of 11 variables in three blocks was as follows:

**Block 1 (control variables):**

1. mHealth app use (dummy-coded)

2. Having a chronic illness or enduring complaints (dummy-coded)

3. Stress symptoms

4. Stress due to overload

**Block 2 (mHealth-related attitudes/affect):**

5. mHealth-related attitudes

6. Skepticism/perceived risks

7. Anxiety toward use

**Block 3 (classic UTAUT (Unified Theory of Acceptance and Use Technology) determinants):**

8. Performance expectancy

9. Effort expectancy

10. Social influence

11. Facilitating conditions

### Main Results

#### Primary Outcome: Determinants of the Acceptance of Stress Management Apps

The significant hierarchical stepwise regression model (Table 3) included 4 of 11 eligible variables from two of three blocks in four steps (*F*_4,136_=56.28, *P*<.001). There was no sign of severe multicolinearity (Durbin-Watson statistic=1.91). The explained variance was 62% in the final step 4 (*R*^2^=.62, *F*_1,136_=6.26, *P*=.01, f^2^=1.63), whereas attitude entered in block 2 (step 3, Table 3) alone added 46% (large effect of f^2^=0.85) after accounting for the influence of the control variables of block 1 (steps 1 and 2, Table 2). Effect sizes for *R*^2^ increase were small to moderate for stress symptoms (f^2^=0.12) and skepticism/perceived risk (f^2^=0.16).

As shown in Table 4, three of four predictors of acceptance remained significant in final step 4: attitude toward using mHealth was the strongest predictor (step 3, beta=0.69, *P*<.001) followed by skepticism/perceived risks (step 4, beta=−0.14, *P*=.01) and stress symptoms (step 2, beta=0.12, *P*=.03). Prior use of mHealth apps became insignificant (beta=0.04, *P*=.54) after accounting for the influence of skepticism/perceived risks. None of the UTAUT predictors (entered as block 3) added a predictive contribution to acceptance after accounting for the influence of attitudes (entered in block 2). Group differences in acceptance ratings based on mHealth use experience are presented in Textbox S1 of Multimedia Appendix 2.

**Figure 2:**
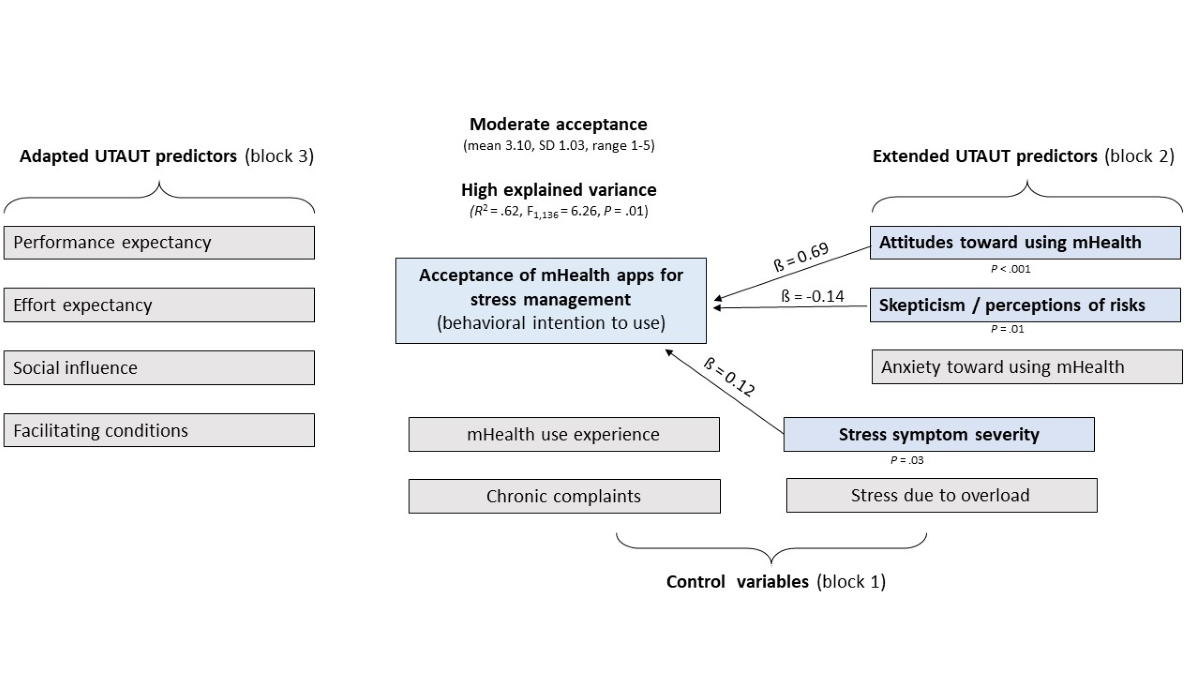
Main findings of the stepwise regression model on the determinants of the acceptance of stress management apps. mHealth: mobile health; UTAUT: Unified Theory of Acceptance and Use of Technology.

Figure 2 shows a summary of the main findings of the primary outcome.

**Table 3:** Model summary of the hierarchical stepwise regression analysis on predictors of the acceptance of stress management apps (N=141).

Model 1^a^	*R*	*R* ^2^	Adjusted *R*^2^	SE	Change in *R*^2^	Change in *F* (df1,df2)	*P* value
Step 1^b^	.32^a^	.10	.10	.98	.10	16.18 (1,139)	<.001
Step 2^c^	.38^b^	.14	.13	.96	.04	6.23 (1,138)	.01
Step 3^d^	.78^c^	.61	.60	.65	.46	161.04 (1,137)	<.001
Step 4^e^	.79^d^	.62	.61	.64	.02	6.26 (1,136)	.01

^a^Dependent variable: acceptance of mobile health (mHealth; behavioral use intention). Model 1 refers to the main model according to the statistical plan in distinction to post hoc analyses. (Models 2 and 3 as presented in Multimedia Appendix 2).

^b^Predictors: (constant), mHealth app use (entered in block 1).

^c^Predictors: (constant), mHealth app use, stress symptoms (block 1).

^d^Predictors: (constant), mHealth app use, stress symptoms (block 1), attitude toward using mHealth (block 2).

^e^Predictors: (constant), mHealth app use, stress symptoms (block 1), attitude toward using mHealth, skepticism/perceived risks (block 2). The UTAUT determinants (entered as block 3) added no further significant predictive contribution and were thus excluded.

**Table 4:** Coefficients of the hierarchical stepwise regression analysis (N=141).

Model 1 and step^a^		Unstandardized coefficient B (SE)	Standardized beta (β)	*P* value	95% CI
**Step 1**					
	(Constant)	2.78 (0.12)	—^b^	<.001	2.55, 3.01
	Use of mHealth^c^ apps (yes)	0.66 (0.17)	0.32	<.001	0.34, 0.99
**Step 2**					
	(Constant)	2.13 (0.28)	—	<.001	1.57, 2.69
	Use of mHealth apps (yes)	0.59 (0.16)	0.29	<.001	0.26, 0.91
	Stress symptoms	0.35 (0.14)	0.20	.01	0.07, 0.62
**Step 3**					
	(Constant)	−0.10 (0.26)	—	.72	−0.61, 0.42
	Use of mHealth apps (yes)	0.10 (0.12)	0.05	.42	−0.14, 0.33
	Stress symptoms	0.18 (0.10)	0.10	.06	−0.01, 0.37
	Attitude toward mHealth	0.84 (0.07)	0.73	<.001	0.71, 0.97
**Step 4**					
	(Constant)	0.52 (0.36)	—	.14	−0.18, 1.22
	Use of mHealth apps (yes)	0.07 (0.12)	0.04	.54	−0.16, 0.30
	Stress symptoms	0.21 (0.09)	0.12	.03	0.03, 0.40
	Attitude toward mHealth	0.78 (0.07)	0.69	<.001	0.65, 0.92
	Skepticism/perceived risks	−0.17 (0.07)	-0.14	.01	−0.31, −0.04

^a^Dependent variable: acceptance of mHealth (behavioral use intention). Model 1 refers to the main model according to the statistical plan in distinction to post hoc analyses. (Models 2 and 3 as presented in Multimedia Appendix 1).

^b^Not applicable.

^c^mHealth: mobile health.

Additionally, a post hoc hierarchical analysis with all 11 variables (see Textbox 2) was performed, with the inclusion method instead of the stepwise method for entering the variables. When all 11 predictors (Multimedia Appendix 2, Tables S4 and S5, model 2) were included, the total *R*^2^ or explained variance was at 64% and thus marginally higher (2%) than for the study model with four variables or steps (62%, see Table 3). In this overall significant post hoc model (*F*_11,129_=20.75, *P*<.001), the increase of explained variance of 1% (*R*^2^=.01) added by the four UTAUT variables in block 3 was not significant (*P*=.59).

Another post hoc hierarchical stepwise regression analysis showed the added predictive value of both positive and negative attitude constructs beyond the UTAUT variables. In contrast to the insignificant contribution UTAUT variables and their exclusion from model 1 as block 3 (Table 3), attitudes and skepticism/perceived risks significantly added explained variance (*R*^2^ increase=.13) when entered as block 3 (Multimedia Appendix 2, Table S6, model 3) beyond three significant UTAUT variables (performance expectancy, facilitating conditions, and social influence). This post hoc model was significant (*F*_7,133_=32.48, *P*<.001). It included seven variables or steps and explained 1% more total variance (*R*^2^=.63) than the study model with four variables or steps (*R*^2^=.62, Table 3). The increase of explained variance of the UTAUT variables after accounting for the control variables was 35% (performance expectancy with 28% as step 3, facilitating conditions with 4% and social influence with 3%) and therefore lower than for attitudes alone (46% *R*^2^ increase) in the study model (see model 1 in Table 3). With the inclusion of attitudes in step 6, all UTAUT variables became insignificant (*P*>.05) and remained so in the final step 7 after entering skepticism/perceived risks (Multimedia Appendix 2, Table S7, models 1-3). Table S8 of Multimedia Appendix 2 shows a summary of all three regression models.

#### Research Question: Influence of Scientific Claims on Mobile Health Acceptance Ratings

The *t* test showed no significant differences in acceptance between participants who read the app description both with (group 1: 70/141, 49.6%; mean 3.37, SD 0.95) and without (group 2: 71/141, 50.4%; mean 3.32, SD 0.86) supplemented scientific claims (*t*_139_=0.31, *P*=.80; Cohen *d*=0.06). Furthermore, there were no significant differences between the two information groups regarding the four UTAUT determinants, attitudes, skepticism/perceived risks, and anxiety (all *P*>.05).

### Secondary Outcome: Preference for and Readiness to Use Mobile Health for Stress Coping

As shown in Table 5, mHealth apps were preferred over medication, a psychiatrist, online counseling, online self-help training, and face-to-face group courses. No differences in the likelihood of future use were identified between mHealth apps versus self-help literature, psychologists, and GPs. Only health information websites were preferred over mHealth apps (*P*<.001).

**Table 5:** Preference for mobile health (mHealth): the likelihood of future use of mHealth apps for stress-related purposes in comparison with other mental health service types (N=141). Dependent variable: likelihood of future use in case of emotional distress (range: 1=very unlikely to 5=very likely).

Service type	Mean (SD)	Mean difference versus mHealth apps (SD)	SE of mean difference	95% CI	*t* (df)	*P* value
mHealth apps	2.67 (1.26)	—^a^	—	—	—	—
Health information website	3.07 (1.29)	−0.40 (1.12)	0.09	−0.58, −0.21	−4.21 (140)	<.001
Online self-help training (ie, computer- and internet-based)	2.45 (1.26)	0.22 (1.18)	0.10	0.02, 0.42	2.21 (140)	.03
Online counseling	2.20 (1.17)	0.48 (1.11)	0.09	0.29, 0.66	5.10 (140)	<.001
Self-help literature	2.73 (1.40)	−0.06 (1.60)	0.13	−0.31, 0.19	−0.45 (140)	.65
Psychologist (therapist or counselor)	2.67 (1.31)	0.01 (1.75)	0.15	−0.28, 0.30	0.05 (140)	.96
Psychiatrist	2.13 (1.07)	0.54 (1.60)	0.13	0.27, 0.81	4.01 (140)	<.001
General practitioner	2.67 (1.27)	0.00 (1.66)	0.14	−0.28, 0.28	0.00 (140)	>.99
Medication-assisted treatment	1.90 (1.10)	0.77 (1.43)	0.12	0.54, 1.01	6.41 (140)	<.001
On-site group course (face-to-face)	2.16 (1.18)	0.51 (1.33)	0.11	0.29, 0.73	4.54 (140)	<.001

^a^Not applicable.

## Discussion

### Principal Results

This study explored the determinants of public acceptance of stress management apps before their integration into statutory health services and the general preferences for mHealth apps compared to other mental health services.

This study indicates a moderate public acceptance of stress management apps in an online sample of German-speaking adults. Considering the relatively early stage of the adoption of e-mental health services in German health care [97], acceptance of mHealth apps probably varies largely based on individual experiences and target populations. The sampling method in this study and the focus on health promotion instead of treatment or aftercare need to be considered as potential reasons for higher acceptance of e-mental health services compared with research in more heterogeneous patient populations [34,44,45] and similar earlier online surveys with nonclinical samples [33,82,83].

#### Determinants of the Acceptance of Stress Management Apps

As a main finding, we identified positive affect or attitude toward using mHealth, skepticism or perceived risk of mHealth (negative cognitive attitudes), and the severity of stress symptoms as significant determinants of the acceptance of mHealth for stress coping. The high magnitude of explained variance of 62% according to Cohen criteria [90,96] is equivalent to other studies on the acceptance of e-mental health services focusing on classic UTAUT determinants, which did not consider attitudes (eg, [34]).

Our main results substantiate research evidence on the key role of attitudes in shaping eHealth and mHealth service acceptance in particular [58,63,98]. Post hoc analysis showed that attitudes and skepticism still added explained variance beyond the control variables and UTAUT determinants, whereas the more elaborated beliefs of UTAUT determinants failed to add a predictive contribution beyond attitudes. This finding can be interpreted in the context of other research on attitude formation and behavioral intentions, which indicated that different levels of elaboration likelihood among end users should be taken into account in early stages of mHealth adoption [99]. For instance, Chen et al [99] showed a moderating effect of privacy concerns on the influence of both perceived usefulness (central route) and trust (peripheral route) on the continuance intention of mHealth apps in a developing market. In our study, the uncertain motivation (low stress levels) and insufficient abilities or knowledge to evaluate mHealth-related questions (nearly half of our sample did not have any mHealth experience) could have yielded a lower elaboration likelihood (peripheral route) reflected by rather undecided views (moderate ratings, tendency toward the middle) and skepticism. In other words, positive attitudes in the sense of an early affective form of opinion formation (regardless of specific knowledge or experience) may be a more relevant initial precondition of acceptance than elaborated cognitive beliefs on usefulness or usability.

Attitudes toward using mobile phones for mental health purposes can differ regarding specific design features or functions [64]; therefore, upcoming surveys could investigate relationships between attitudes, beliefs, and acceptance with respect to distinct components and functionalities of available stress management apps. Among other components, perceived value by users, visual design, usability, the potential to improve user engagement, tailoring and personalization, gratification, and information and content have been suggested as key drivers of the real-world uptake and user retention in eHealth and mHealth interventions (eg, [100-103]). These cannot be evaluated with the predictive acceptance model we applied. Nonetheless, our results on the major role of attitudes in mHealth acceptance provide implications on aspects to consider in practice. For instance, future efforts aiming at improving the adoption of e-mental health services could put emphasis on the pleasant or joyful aspects of using apps. For example, the yet not fully utilized potential of gamification for supporting the acquisition of behavior change techniques could be promoted as a clear benefit of mobile versus Web-based stress management programs [104].

Effective interventions to increase user retention in mental health services usually involve a comprehensive approach targeting attitudes, knowledge, needs, and barriers [105]. As a relevant barrier, our findings confirmed the negative influence of skepticism and perceptions for stress management apps, which complements findings from clinical settings (eg, [61]) and a recent meta-analysis [63], showing that both attitudes and perceived risks are determinants of the behavioral intention to use mHealth apps. Trustworthiness, data security, and privacy are main issues raised by consumers [102] and health professionals [106]. It is important to address concerns and misconceptions with acceptance-facilitating interventions, as effectively demonstrated by RCTs in different German health care settings (eg, [43,45]). Fostering positive attitudes toward mHealth would also be important in the context of the workplace, which is a common source of stress and stress-related disorders [107]. In accordance with social influences on the acceptance of health services, research indicates a higher interest in using apps for workplace health promotion among leaders with positive attitudes [9]. Therefore, health professionals and other multipliers and stakeholders should be involved in the dissemination of mental health apps.

Furthermore, personal relevance and mental health needs may affect the acceptance of mHealth apps; therefore, public health initiatives on mHealth could highlight the benefits of preventive innovations that tend to diffuse very slowly (delay of reward after adoption), as proposed by Rogers [108]. Although our results correspond to findings on the relationship between stress and interest in using mHealth for stress management [75], it is important to mention that self-reported stress severity in our sample was low to moderate. Considering that the main target group for primary prevention and health promotion in Germany is healthy adults [109], such apps may have the highest potential to reach populations that are already rather privileged in terms of having the necessary resources and knowledge to efficiently use mental health services, as was the case in our sample. The challenge is to increase the uptake of self-help tools in populations that are traditionally hard to reach and among those with mental health needs who are unlikely to use psychological services [32,62].

Considering the positive influence of personal experience (with mobile phones [110] and/or mHealth [69,100]) on the acceptability or uptake of mHealth apps, our findings support the suggestion to increase the availability of expert-guided possibilities for consumers or patients to try quality-approved apps. This would require making mental health professionals familiar with such services since prior research has shown personal use experience as a driver for use in their practice [111].

#### Influence of Information Cues in an Exemplary App Description

Beyond the identification of determinants of acceptance, to our knowledge, our study was one of the first to explore the influence of scientific claims on consumer acceptance of a hypothetical app. Keeping the elaboration likelihood model in mind, the fact that we found no difference to the group receiving basic information only is somewhat consistent with the major role of attitudes in our study, the very low awareness of e-mental health treatments, and the moderate level of mHealth experience. However, it is also possible that vague scientific claims were not persuasive for a selective, overall well-educated sample of mobile phone users, considering that the reputation or credibility of the source of information cues were shown as a relevant factor in the formation of attitudes and use intentions of e-mental health and mHealth services (eg, [59,99]). Overall, the main issues may be that the text we used for both vignettes was created based on modified information from a website on a digital stress coping program for university students (academic audience) and the variances between both vignettes (content and length) were too small to find a significant difference.

Accordingly, quality of content and validity of information have been identified as important domains for the real-world uptake of mHealth apps [102]. However, the evidence base for the quality and efficacy of most mental health apps is limited [112], even among those mental health apps that claim to be effective [113]. Importantly, a study by Schueller et al [69] showed that perceived usefulness of mental health apps rated by consumers is not necessarily equivalent to what the research evidence suggests. The influence of perceived credibility by users could be another option for surveys on the acceptance of e-mental health studies [88,114].

#### Preference for and Readiness to Use Mobile Health for Stress Coping

Another aim was to assess the preference of mHealth for stress coping. We identified preferences for mHealth apps over face-to-face group training, Web-based self-help programs, medication, and consulting psychiatrists. This points to an additional potential of digital or app-based courses versus traditional face-to-face group courses in primary prevention in reaching further populations that are not severely stressed and are familiar with using mobile phones. Wahbeh et al [115] showed a preference for a Web-based over group format for mindfulness interventions. That study and our findings show that online recruitment should be considered as a potential reason for a higher preference of e-mental health services compared to more diverse samples in health care. However, the lower interest in using Web-based than app-delivered self-help programs contrasts with findings from an Australian study by Batterham and Calear [62]. A possible explanation is that our study was conducted in an environment where eHealth or mHealth availability in German routine care—and thus adoption—is still in an earlier stage than in other European countries such as Sweden (eg, [116-118]). In contrast, mHealth apps can be downloaded by everyone and used outside of health care. Hence, we assume that our online sample of German-speaking participants was overall less familiar because German-speaking countries (ie, Germany, Austria and some regions in Switzerland) less often have openly accessible Web-based psychological programs available than publicly available mHealth apps. In comparison, countries such as Australia (eg, [119]) have such Web-based programs already established and available for the public. This issue is reflected by the very low awareness of e-mental health therapies (21%) in our sample and in other online surveys [33,59,74] and a German panel survey (SOEP-IS innovative modules, internet-based psychotherapy, [94] written communication with Apolinário-Hagen J, unpublished raw data, 2016). Another reason might be that, for health promotion purposes, app-delivered programs may be seen as more convenient to use in daily life [83].

Contrary to prior research considering clinically relevant mental health issues (eg, [83,86]), we found no difference in the readiness to use mHealth apps in comparison with services provided by psychologists and self-help literature for stress-related purposes. Potentially, participants in our study viewed stress as a usual, rather mild issue that can be better addressed through different ways of self-help (with or without psychological support) than with clinical interventions or through medical support.

Consequently, the highest likelihood of use was found for health information websites for stress-related purposes, as already shown in other German studies [34,84]. A possible reason is that health information websites are self-help options with the lowest barrier to access because they can be retrieved publicly with several devices (eg, desktop computer, tablet, mobile phone), are usually free of cost and do not require downloading another app and/or any registration. In this sense, “Dr. Google” enables tailored advice for mental health purposes on demand, which may explain their high acceptance [86]. Likewise, a qualitative study [120] showed that employees characterized an optimal e-mental health intervention as a website with interactive elements that involve temporarily unlimited access to state-of-the-art information and advice.

There are several initiatives providing guidance on e-mental health and mHealth quality criteria and certification (eg, [27,103]), but such information should be connected with certified services and brought to the awareness of more consumers and health professionals. Information websites in the sense of a low-threshold public health service could provide evidence-based information on stress prevention, stress screening tools, and access to mHealth apps. Psychoeducational information could be used to improve e-mental health literacy, which would help improve help-seeking intentions and behavior [121] or could be integrated into a stepped care prevention approach [122]. Textbox 3 shows the main findings and implications of our study.

**Summary of key findings and novel insights.**

**What this study shows that was already known:**

- Attitudes are a key determinant of behavioral intention to use mobile health (mHealth)
- The low rates of awareness and use of electronic mental (e-mental) health treatments in our sample are in line with findings from other online surveys and panel surveys from Germany
- Skepticism and perception of risks (eg, privacy) are important barriers for e-mental health and mHealth acceptance
- Perceived stress needs further consideration in mHealth acceptance models
- Preference is for information websites over (less accessible) mental health services for stress prevention, including mHealth apps and face-to-face group interventions

**Which novel implications and insights this study adds to the research evidence:**

- Moderate and slightly higher acceptance of mental health apps and e-mental health services compared with other online surveys with community samples and studies with patient populations (implication: scope on health promotion and stress prevention rather than on treatment with disorder-specific focus or clinical wording for e-mental health programs)
- Unified Theory of Acceptance and Use Technology (UTAUT) studies on acceptance of mental health apps should consider attitudes and more elaborated beliefs (implication: adaptations of predictive acceptance models across stages of diffusion of mHealth adoption)
- Preference for mHealth over Web-based programs and state-of-the-art group stress management programs (implication: outline specific benefits of mHealth for stress management to use in daily life, but also educate about the potentials of Web-based and face-to-face courses)
- Comparable preferences for mHealth and traditional psychological services (implication: provide a set of choices tailored to individual needs and preferences)

### Limitations

The exploratory nature of our study has several limitations to be considered when interpreting the findings.

First, the online recruitment and sample size limit the generalizability of our results; therefore, we cannot draw conclusions for the general German population. Also, the focus of this study on the public acceptance of stress coping apps and health promotion, and the necessary slight adaptations of some scales to the mHealth context, impede the comparability with most studies in this field that targeted e-mental health treatments [33,82,83] or specific mental disorders, such as depression [43,123,124]. In addition, due to the absence of norm values, we classified acceptance as moderate based on prior work [34]; therefore, it is debatable whether the acceptance was really moderate (external validity).

Second, the subjective stress level in this sample was relatively low, with a mean sum score of 26.63 (SD 7.71) compared with the norm sample in the SCI test manual (mean 34.07, SD 7.96, possible range 13-65). Also, the selective sample of 96% mobile phone users of mostly young and higher educated adults (more than 60% with academic degree) may further explain the moderate acceptance of mHealth. A next step could be to compare the acceptance of e-mental health services in samples with different stress levels. To overcome the self-selection bias, recruitment in primary care with referrals from GPs could be an option.

Third, the lack of a passive control condition makes it impossible to state whether the information about an exemplary app may have biased the acceptance ratings toward more positive ratings (eg, [59]). Future studies should control for the impact of information about real, well-known apps on acceptance using a pre-post design and a manipulation or intervention check before implementation. We have also applied a similar, but more elaborated, approach with text-based information on two existing evidence-based programs for stress coping (StudiCare for students and GET-ON for employees) in winter 2018 and found that the majority did not know these programs [88]. Therefore, it is debatable whether it would have made a difference to name an existing program in our sample. Potentially, as previously outlined, it would be another option to test our hypotheses with freely available commercial apps with the highest download rates, although this would impede the assessment of the general acceptance of mHealth apps.

Fourth, formulating expectations on a fictional app was likely to be difficult compared with rating an app that is known to the participants or has been used already, as the feedback from our participants suggested. Furthermore, of 230 participants who started the survey, 89 dropped out (half of them after the first UTAUT questions). In addition, the differences between both vignettes were a few abstract sentences including vague information on the effectiveness of a hypothetical app. Since the text of both vignettes was adapted from a website that recruited distance-learning students for a RCT on the effectiveness of an evidence-based digital stress intervention, the content may have been rather academic or too abstract for the broader population targeted in our study. Therefore, this experimental approach may have been too artificial (eg, questionable content validity) and be the main reason for finding no group differences.

Fifth, the readiness to use mHealth was assessed without standardized information on service types, similar to “real-world” help-seeking situations. In addition, we did not ask for what the participants understood under each service. Finally, similar to most UTAUT studies [49,125], we used a cross-sectional study design with acceptance as the dependent variable. This cannot address the well-known problem of the intention-behavior gap (eg, [126]), in which attitude strength related to personal relevance and experience has been suggested as a factor to bridge this gap in technology use [127]. Hence, our findings should be seen as preliminary and interpreted with caution.

### Conclusions

Attitudes may play a pivotal role in shaping public acceptance toward stress management apps in an early stage of the adoption of e-mental health services. Concerns regarding the use of apps for stress management purposes could be addressed through health information websites and public health campaigns that can help increase knowledge about the benefits of stress prevention and information on mental health services.

## Appendix

Multimedia Appendix 1 Methods supplementary data.

Multimedia Appendix 2 Results supplementary data.

## Abbreviations

GP: general practitioner
mHealth: mobile health
RCT: randomized controlled trial
SCI: Stress and Coping Inventory
UTAUT: Unified Theory of Acceptance and Use Technology
