# Supplementary material for: Determinant Factors of Public Acceptance of Stress Management Apps: Survey Study
Source: JMIR Ment Health. 2019 Nov 7;6(11):e15373. doi: 10.2196/15373 (PMC6873149; doi:10.2196/15373)
Supplement: Multimedia Appendix 1 [file mental_v6i11e15373_app1.pdf]

Multimedia Appendix 1

Table S1. Adapted items of the UTAUT model and related extended predictors with references of original publications[a -f].

Questionnaire	Scale	Items – English translation (German original)
**UTAUT** (main scales)[a]	Behavioral intention to use (acceptance pf mHealth for stress management or coping purposes)	1. I would like to try “COPE”. (Ich würde "COPE" gerne ausprobieren.)[b,c]<br>2. I would use “COPE” if it were offered to me. (Ich würde "COPE" verwenden, wenn es mir angeboten würde.)[b,c]<br>3. For me, “COPE” would be worth paying for. (Es wäre mir wert, für "COPE" zu bezahlen.)[b,c]
	Performance expectancy	1. “COPE” could help me improve my work-related well-being. (“COPE" könnte mir helfen, mein arbeitsbezogenenes Wohlbefinden zu verbessern.)[b,c]<br>2. “COPE” could help me with work-related stress. (“COPE“ könnte mir bei arbeitsbezogenem Stress helfen.)[b,c]<br>3. “COPE” could help me improve my personal health. (“COPE“ könnte meine persönliche Gesundheit verbessern.)[b,c]<br>4. The app [COPE] could help me increase my chances of better managing stress. (Mit Hilfe der App kann ich die Chancen erhöhen, besser mit Stress umzugehen.)[b,c,d]
	Effort expectancy	1. I imagine the app would be clear and understandable. (Die Bedienung der App stelle ich mir klar und verständlich vor.)[a,c,e]<br>2. I could integrate “COPE” in my everyday life. (Ich könnte “COPE“ in mein tägliches Leben integrieren.)[b,c]<br>3. It will be easy to use the app. (Es wird einfach sein, die App zu bedienen.)[a,c,e]<br>4. “COPE“ would be easy to understand and use. (“COPE“ wäre einfach zu verstehen und zu bedienen).[a,b,c,e]
	Social influence	1. People close to me would approve of “COPE”. (Personen in meinem engeren Umfeld würden “COPE“ befürworten.)[b,c]<br>2. My friends would approve of “COPE”. (Meine Freunde würden “COPE“ befürworten.)[b,c]<br>3. My general practitioner would approve the use of “COPE”. (Mein Hausarzt würde die Verwendung von “COPE“ befürworten.)[b,c]
	Facilitating conditions	1. I have the necessary technical preconditions for using “COPE“. (Ich besitze die technischen Voraussetzungen um "COPE" verwenden zu können.)[b,c]<br>2. I possess the technical know-how to utilize “COPE”. (Ich besitze das technische „Know-How“, um "COPE" nutzen zu können.)[b,c]
**Attitude/affect toward using technology** (UTAUT-related dropped scale)[a,e]	Attitude toward using mHealth for stress management / coping	1. Using the app is a good idea. (Es ist eine gute Idee, die App zu nutzen.)[a,e]<br>2. Using the app would be interesting. (Es wäre interessant die App zu nutzen.)[a,e]<br>3. Using the app could be fun. (Die Nutzung der App könnte Spaß machen.)[a,e]<br>4. I would like to work with the app. (Ich würde es mögen, mit der App zu arbeiten.)[a,e]
**Anxiety toward using technology** (UTAUT-related dropped scale)[a,e]	Anxiety toward using mHealth for stress management / coping	1. I feel apprehensive about using the app. (Ich habe Bedenken, was die Nutzung der App betrifft.)[a,e]<br>2. It scares me to imagine that I could lose a lot of information by hitting the wrong key. (Es macht mir Angst, mir vorzustellen, durch das Drücken einer falschen Taste, Informationen zu verlieren.)[a,e]<br>3. I hesitate to use the app out of fear of making mistakes I cannot correct. (Ich zögere die App zu nutzen, aus Angst Fehler zu machen, die man nicht korrigieren kann.)[a,e]

		4. The app is somewhat intimidating to me. (Die App schüchtert mich etwas ein.)[a,e]
**APOI** (subscale)[f,g]	Skepticism and perception of risks	1. When using an app for stress reduction, I do not expect long-term effects. (Wenn ich eine App zur Stressreduktion nutze, erwarte ich keine Langzeiteffekte.)[f,g]<br>2. When using an app for stress reduction, I do not receive professional support. (Wenn ich eine App zur Stressreduktion nutze, bekomme ich keine professionelle Unterstützung.)[f,g]<br>3. It will be difficult to implement the suggestions of an app for stress reduction effectively into my everyday life. (Ich werde Schwierigkeiten haben, die Vorschläge der App zur Stressreduktion in meinem täglichen Leben umzusetzen.)[f,g]

[a]Unified theory of acceptance and use of technology (UTAUT; Venkatesh et al., 2003).

[b]The German UTAUT items were adapted based on prior research (Hennemann et al., 2016). The term "Web-based aftercare" was replaced by "app" or "COPE" (i.e., the exemplary app "COPE" that was presented in the randomized subsection of the survey).

[c]The wording of the UTAUT items was tailored to "app" or "COPE (i.e., the exemplary app "COPE" that was presented in the randomized subsection of the survey).

[d]The term "health problems" (Hennemann et al., 2016) was replaced by "better managing stress".

[e]Adapted to the mHealth context based on items from the original UTAUT study (Venkatesh et al., 2003).

[f]Attitudes towards Psychological Online Interventions (APOI; Schröder et al., 2015).

[g]The German APOI items of the subscale skepticism and perception of risks (Schröder et al., 2015) were adapted to the app/mHealth context. Psychological online intervention (POI) was replaced by the term "app" and extended with the purpose of "stress reduction".
