# Supplementary material for: Determinant Factors of Public Acceptance of Stress Management Apps: Survey Study
Source: JMIR Ment Health. 2019 Nov 7;6(11):e15373. doi: 10.2196/15373 (PMC6873149; doi:10.2196/15373)
Supplement: Multimedia Appendix 2 [file mental_v6i11e15373_app2.pdf]

Multimedia Appendix 2

Descriptive data on chronic complaints

Table S2. Descriptive data on chronic illness or enduring/recurrent complaints for more than 3 weeks (n=41)[a].

**Chronic illness or enduring/recurrent complaint**	**N=41**	**N=141**
Upper or lower back pain, n (%)	20 (48.8)	(14.2)
Neck pain	14 (34.1)	(9.9)
Musculoskeletal disorders (e.g., herniated disc, hernias, arthritis)	9 (22.0)	(6.4)
Migraine	8 (19.5)	(5.7)
Chronic obstructive pulmonary disease	1 (2.4)	(0.7)
Iron deficiency anemia	2 (4.9)	(1.4)
Depression	11 (26.8)	(7.8)
Anxiety / anxiety disorder	4 (9.8)	(2.8)
Other mental disorder (excl. anxiety and depression)	4 (9.8)	(2.8)
Diabetes	0	0
Loss of hearing	0	0
**Other (commentary section)**		
Asthma	1 (2.4)	(0.7)
Stomach pain	2 (4.9)	(1.4)
Chronic autoimmune disease	1 (2.4)	(0.7)
Diarrhea	1 (2.4)	(0.7)
Hashimoto	1 (2.4)	(0.7)
Hypertension	1 (2.4)	(0.7)
Knee (complaints)	1 (2.4)	(0.7)
Food allergies	1 (2.4)	(0.7)
Psoriasis	1 (2.4)	(0.7)
Neurodermatitis	1 (2.4)	(0.7)
Heartburn	1 (2.4)	(0.7)
Metabolic disorder	1 (2.4)	(0.7)
Teeth (dental problems)	1 (2.4)	(0.7)

[a] Multiple answers were possible.

Preliminary analyses for the primary outcome

Table S3. Descriptive data of variables and correlations between acceptance and the predictors (N =141) [a-c].

Variable		N = 141
**Metric variables**	**M (SD), range of scale** (observed range)[a]	**Correlation with acceptance** (coefficient r)[b]
Criterion		
Acceptance/behavioral use intention	3.10 (1.03), 1-5 (1-5)	
Adapted UTAUT predictors		
Performance expectancy	3.11 (0.89), 1-5 (1-5)	.64[c]
Effort expectancy	3.68 (0.81), 1-5 (1-5)	.46[c]
Social influence	3.01 (0.74), 1-5 (1-4.67)	.49[c]
Facilitating conditions	4.25 (0.89), 1-5 (1-5)	.28[c]
Extended predictors (metric)		
Attitude toward using mHealth	3.44 (0.90), 1-5 (1-5)	.77[c]
Anxiety toward using mHealth	2.03 (0.85), 1-5 (1-4)	-.22[c]
Skepticism/perceived risks	2.83 (0.85), 1-5 (1-5)	-.38[c]
Stress due to permanent Smartphone/mobile phone availability (single item)	3.01 (1.27), 1-5 (1-5)	-.06
Stress symptoms (severity, past 6 months), mean score,<br>Mean sum score	1.97 (0.59), 1-5 (1-3.54)<br>26.63 (7.71), 13-46	.25[c]
Stress due to overload (past 3 months)	3.58 (1.26), 1-7 (1-7)	.21[c]
Positive thinking (stress coping)	2.63 (0.56), 1-4 (1-4)	-0.9
Active coping (stress coping)	2.54 (0.66), 1-4 (1-4)	.05
Social support (stress coping)	3.19 (0.68), 1-4 (1.5-4)	.15
Increased alcohol/cigarettes consumption	1.68 (0.79), 1-4 (0.75-3.75)	-.05
eHealth literacy	3.72 (0.73), 1-5 (1.25-5)	.04
Age (years)	34.84 (11.09), 19-76	-.07
**Categorial variables**	**Number of categories** (n)	**Correlation with acceptance** (coefficient r)[b]
Extended predictors (categorial)		
Gender (male, female)	2	-.03
Education level (none or low-high)	10	-.01
Having a chronic illness or enduring complaints (yes, no)	2	-.23[c]
Use of Smartphone (familiarity with use; yes, no)	2	.06
Prior use of mHealth app use (yes, no) – filter question	2	-.32[c]

Frequency of using mHealth apps (once per month or less – daily)	5	.11
Duration of mHealth app use (since few weeks – more than 2 years)	5	.09
Awareness of internet therapies (yes, no)	2	-.01
Prior use of internet therapies (yes, no)	2	-.18

[a]All items of the metric scales were assessed on Likert scales, with higher scores indicating a higher agreement to presented statements. The measures are presented in the methods section of the main text of this paper. A deviation from the fixed scale range to the observed range is possible for scales with a computed mean score and for with items that had to be recoded, such as those of the Stress-Coping-Inventory (SCI; Satow, 2012).

[b]Pearson´s correlation coefficient r. Only variables with significant zero-order correlation with acceptance mean score ($P < .05$) were included in the hierarchical regression analysis.

[c] * $P < .05$; Based on significant correlations with acceptance, 11 out of 25 variables were included in the hierarchical regression analysis as predictors of acceptance.

Post-hoc analyses for the primary outcome

Textbox S1. Significant group differences in acceptance and its determinants between participants with (n=69) and without experience (n=71) with any kind of mHealth apps (N=141).

- Significantly higher scores among participants with any kind of mHealth experience (48.9% vs. 51.1%) were found for acceptance (Welch, $t_{(137,532)}$=4.04, P<.001), and its identified determinants attitude (Welch, $t_{(129,123)}$=4.45, P<.001), and stress symptoms (Welch, $t_{(130,666)}$=2.18, P=.03), while skepticism/perceived risks was lower compared to those without mHealth experience ($t_{(139)}$=-2.12, P=.04).
- Furthermore, higher scores in the group with mHealth experience were found for three out of four of the UTAUT determinants: performance expectancy (Welch, $t_{(134,146)}$=3.92, P<.001), effort expectancy ($t_{(139)}$=2.86, P=.01), and social influence ($t_{(139)}$=2.41, P=.02).

Post-hoc hierarchical regression analysis with inclusion method (Model 2)

Table S4. Model summary of the post-hoc hierarchical regression analysis on predictors of the acceptance of stress management apps and the added predictive value of UTAUT variables using inclusion method for entering 11 variables in three blocks (N=141)[,a-d].

Model 2[a]	R	R^{2e}	adjusted R^2	SE	change in R^2	change in F	df1	df2	P-value
Block 1[b]	.40[a]	.16	.13	.96	.16	6.38	4	136	<.001
Block 2[c]	.79[b]	.63	.61	.64	.47	56.84	3	133	<.001
Block 3[d]	.80[c]	.64	.61	.64	.01	0.70	4	129	.59

a. Dependent variable: acceptance of mHealth (behavioral use intention). Model 2 refers to the first post-hoc analysis in distinction to the study model from the statistical plan (Model 1) and the post-hoc analysis using the stepwise method for entering variables in the hierarchical regression analysis to assess the added predictive contribution of attitude constructs beyond the UTAUT variables (Model 2).
b. Predictors: (constant), mHealth app use, stress symptoms (block 1)
c. Predictors: (constant), mHealth app use, stress symptoms (block 1), attitudes toward using mHealth, skepticism/perceived risks (block 2)

d. Predictors: (constant), mHealth app use, stress symptoms (block 1), attitudes toward using mHealth, skepticism/perceived risks (block 2), performance expectancy, facilitating conditions, social influence (block 3)

Table S5. Coefficients of the post-hoc hierarchical stepwise regression analysis on the added predictive value of the UTAUT variables for predicting mHealth acceptance (N=141)[a].

Model 3/block		Unstandardized coefficient B		Standardized beta		
		**B**	**SE**	**Beta (ß)**	**T**	**P-value**
1	(constant)	2.15	.30		7.10	<.001
	Use of mHealth apps (yes)	0.56	.17	0.27	3.37	.001
	Chronic complaints (yes)	0.25	.20	0.11	1.29	.20
	Stress due to overload	0.05	.08	0.06	0.56	.58
	Stress symptoms	0.22	.18	0.13	1.25	.21
2	(constant)	0.70	.39		1.81	.07
	Use of mHealth apps (yes)	0.05	.12	0.02	0.38	.71
	Chronic complaints (yes)	0.19	.13	0.09	1.43	.15
	Stress due to overload	0.02	.06	0.03	0.37	.71
	Stress symptoms	0.16	.12	0.09	1.30	.20
	Attitude toward mHealth	0.76	.07	0.67	10.80	<.001
	Anxiety toward use of mHealth	-0.05	.07	-0.04	-0.64	.52
	Skepticism/perceived risks	-0.18	.07	-0.15	-2.52	.01
3	(constant)	0.40	.54		0.73	.47
	Use of mHealth apps (yes)	0.05	.12	0.02	0.38	.71
	Chronic complaints (yes)	0.21	.14	0.09	1.54	.13
	Stress due to overload	0.01	.05	0.01	0.12	.90
	Stress symptoms	0.14	.13	0.08	1.14	.26
	Attitude toward mHealth	0.67	.11	0.59	6.25	<.001
	Anxiety toward use of mHealth	-0.04	.07	-0.03	-0.51	.61
	Skepticism/perceived risks	-0.16	.07	-0.13	-2.15	.03
	Performance expectancy	0.09	.10	0.07	0.85	.40
	Effort expectancy	-0.04	.09	-0.03	-0.47	.64
	Social influence	0.12	.09	0.09	1.31	.19
	Facilitating conditions	0.03	.08	0.03	0.39	.70

a. Dependent variable: acceptance of mHealth (behavioral use intention). Model 2 refers to the first post-hoc analysis in distinction to the study model from the statistical plan (Model 1) and the post-hoc analysis using the stepwise method for entering variables in the hierarchical regression analysis to assess the added predictive contribution of attitude constructs beyond the UTAUT variables (Model 2).

Post-hoc hierarchical stepwise regression analysis (Model 3)

Table S6. Model summary of the post-hoc hierarchical stepwise regression analysis on predictors of the acceptance of stress management apps on the added value of attitude constructs beyond UTAUT variables (N=141)[a-h].

Model 3[a]	R	R^{2e}	Adjusted R^2	SE	Change in R^2	Change in F	df1	df2	P-value
Step 1[b]	.32	.10	.10	.98	.10	16.18	1	139	<.001
Step 2[c]	.38	.14	.13	.96	.04	6.23	1	138	.01
Step 3[d]	.65	.43	.41	.79	.28	67.63	1	137	<.001
Step 4[e]	.68	.47	.45	.76	.04	10.59	1	136	.01
Step 5[f]	.70	.50	.48	.74	.03	7.56	1	135	.01
Step 6[g]	.79	.62	.60	.65	.12	43.07	1	134	<.001
Step 7[h]	.79	.63	.61	.64	.01	4.48	1	133	.04

a. Dependent variable: acceptance of mHealth (behavioral use intention). Model 3 refers to the second post-hoc analysis in distinction to the study model from the statistical plan (Model 1) and the post-hoc analysis using the inclusion method for entering variables in the hierarchical regression analysis (Model 2).
b. Predictors: (constant), mHealth app use (entered in block 1)
c. Predictors: (constant), mHealth app use, stress symptoms (block 1)
d. Predictors: (constant), mHealth app use, stress symptoms (block 1), performance expectancy (block 2)
e. Predictors: (constant), mHealth app use, stress symptoms (block 1), performance expectancy, facilitating conditions (block 2)
f. Predictors: (constant), mHealth app use, stress symptoms (block 1), performance expectancy, facilitating conditions, social influence (block 2)
g. Predictors: (constant), mHealth app use, stress symptoms (block 1), performance expectancy, facilitating conditions, social influence (block 2), attitudes toward using mHealth (block 3)
h. Predictors: (constant), mHealth app use, stress symptoms (block 1), performance expectancy, facilitating conditions, social influence (block 2), attitudes toward using mHealth, skepticism/perceived risks (block 3)

Table S7. Coefficients of the post-hoc hierarchical stepwise regression analysis on the added value of attitude constructs on acceptance (N=141)[a].

		Unstandardized coefficient B		Standardized beta		
**Model 3/step**		**B**	**SE**	**Beta (ß)**	**T**	**P-value**
1	(constant)	2.78	.12		24.14	<.001
	Use of mHealth apps (yes)	0.66	.17	0.32	4.02	<.001
2	(constant)	2.13	.28		7.54	<.001
	Use of mHealth apps (yes)	0.59	.16	0.29	3.58	<.001
	Stress symptoms	0.35	.14	0.20	2.50	.01
3	(constant)	0.66	.29		2.26	.03
	Use of mHealth apps (yes)	0.26	.14	0.13	1.88	.06
	Stress symptoms	0.13	.12	0.07	1.07	.29
	Performance expectancy	0.66	.08	0.58	8.22	<.001
4	(constant)	-0.43	.44		-0.99	.33
	Use of mHealth apps (yes)	0.25	.14	0.12	1.83	.07
	Stress symptoms	0.24	.12	0.14	2.04	.04
	Performance expectancy	0.60	.08	0.52	7.47	<.001
	Facilitating conditions	0.25	.08	0.23	3,25	.001
5	(constant)	-0.74	.44		-1.67	.10
	Use of mHealth apps (yes)	0.23	.13	0.11	1.74	.09

	Stress symptoms	0.21	.12	0.12	1.84	.07
	Performance expectancy	0.51	.09	0.44	5.92	<.001
	Facilitating conditions	0.21	.08	0.19	2.80	.01
	Social influence	0.27	.10	0.20	2.75	.01
6	(constant)	-0.48	.39		-1.23	.22
	Use of mHealth apps (yes)	0.09	.12	0.04	0.76	.45
	Stress symptoms	0.17	.10	0.10	1.65	.10
	Performance expectancy	0.11	.10	0.09	1.10	,273
	Facilitating conditions	0.04	.07	0.04	0.58	,566
	Social influence	0.14	.09	0.10	1.51	,133
	Attitude toward mHealth	0.69	.11	0.60	6.56	<.001
7	(constant)	0.11	.47		0.22	.82
	Use of mHealth apps (yes)	0.07	.12	0.04	0.61	.55
	Stress symptoms	0.20	.10	0.12	1.98	.049
	Performance expectancy	0.09	.10	0.08	0.90	.37
	Facilitating conditions	0.04	.07	0.04	0.60	.55
	Social influence	0.10	.09	0.07	1.14	.26
	Attitude toward mHealth	0.67	.10	0.59	6.43	<.001
	Skepticism/perceived risks	-0.15	.07	-0.12	-2.12	.04

a. Dependent variable: acceptance of mHealth (behavioral use intention). Model 3 refers to the second post-hoc analysis in distinction to the study model from the statistical plan (Model 1) and the post-hoc analysis using the inclusion method for entering variables in the hierarchical regression analysis (Model 2).

Table S8. Summary of the coefficients of the hierarchical regression analyses of the study model and two post-hoc models (N=141)[a].

**Predictors of mHealth acceptance**	Order of blocks	**Model 1 (study model)** *stepwise method (added value of UTAUT variables)*		**Model 2 (post-hoc analysis)** *inclusion method (added value of UTAUT variables)*		**Model 3 (post-hoc analysis)** *stepwise method (added value of attitude constructs)*		Order of blocks
**Variables**	**Blocks of Model 1+2**	**Variable included in steps** (* sign.)	**Beta (ß)** (in final step 4)	**Variable included in blocks** (* sign.)	**Beta (ß)** (step 3)	**Included in steps**	**Beta (ß)** (in final step 7)	**Blocks of Model 3**
**Control variables**								
Use of mHealth apps		1*, 2*, 3, 4	0.04	1*, 2, 3		1*, 2*, 3, 4, 5, 6, 7		
Having chronic complaints				1, 2, 3				
Stress due to overload				1, 2, 3				
Stress symptoms	Block 1	2*, 3, 4*	0.12	1, 2, 3		2*, 3*, 4*, 5, 6, 7*	0.12	Block 1
**mHealth-related attitudes and beliefs**								
Attitude toward mHealth		3*, 4*	0.69	2*, 3*	0.57	6*, 7*	0.59	
Anxiety toward use of mHealth				2, 3				
Skepticism/perceived risks	Block 2	4*	-0.14	2*, 3*	-1.28	7*	-0.12	Block 3
**UTAUT determinants**								
Performance expectancy				3		3*, 4*, 5*, 6, 7		
Effort expectancy				3				
Social influence				3		5*, 6, 7		
Facilitating conditions	Block 3			3		4*, 5*, 6, 7		Block 2
Total explained variance		**R^2 = .62**		**R^2 = .64**		**R^2 = .63**		

a. Dependent variable: acceptance of mHealth (behavioral use intention). * significant (*P*<.05)
